# Human–Dromedary Camel Interactions and the Risk of Acquiring Zoonotic Middle East Respiratory Syndrome Coronavirus Infection

**DOI:** 10.1111/zph.12171

**Published:** 2014-12-27

**Authors:** C. Gossner, N. Danielson, A. Gervelmeyer, F. Berthe, B. Faye, K. Kaasik Aaslav, C. Adlhoch, H. Zeller, P. Penttinen, D. Coulombier

**Affiliations:** ^1^ European Centre for Disease Prevention and Control (ECDC) Stockholm Sweden; ^2^ School of Public Health and Primary Care (CAPHRI) Maastricht University Medical Center (MUMC+) Maastricht The Netherlands; ^3^ Animal and Plant Health Unit European Food Safety Authority (EFSA) Parma Italy; ^4^ FAO/CIRAD‐ES Campus International de Baillarguet Montpellier France

**Keywords:** Arabian Peninsula, camels, coronavirus, MERS‐CoV, zoonoses

## Abstract

Middle East respiratory syndrome coronavirus (MERS‐CoV) cases without documented contact with another human MERS‐CoV case make up 61% (517/853) of all reported cases. These primary cases are of particular interest for understanding the source(s) and route(s) of transmission and for designing long‐term disease control measures. Dromedary camels are the only animal species for which there is convincing evidence that it is a host species for MERS‐CoV and hence a potential source of human infections. However, only a small proportion of the primary cases have reported contact with camels. Other possible sources and vehicles of infection include food‐borne transmission through consumption of unpasteurized camel milk and raw meat, medicinal use of camel urine and zoonotic transmission from other species. There are critical knowledge gaps around this new disease which can only be closed through traditional field epidemiological investigations and studies designed to test hypothesis regarding sources of infection and risk factors for disease. Since the 1960s, there has been a radical change in dromedary camel farming practices in the Arabian Peninsula with an intensification of the production and a concentration of the production around cities. It is possible that the recent intensification of camel herding in the Arabian Peninsula has increased the virus' reproductive number and attack rate in camel herds while the ‘urbanization’ of camel herding increased the frequency of zoonotic ‘spillover’ infections from camels to humans. It is reasonable to assume, although difficult to measure, that the sensitivity of public health surveillance to detect previously unknown diseases is lower in East Africa than in Saudi Arabia and that sporadic human cases may have gone undetected there.

**Impact**

- Dromedary camels are the only animal species for which there is convincing evidence that it is a host species for MERS‐CoV and hence a potential source of human infections.
- Direct contact with dromedary camels can only explain a small proportion of the primary cases. Other possible sources and vehicles of infection include food‐borne transmission through consumption of unpasteurized camel milk and raw meat, medicinal use of camel urine and zoonotic transmission from other species.
- In the Arabian Peninsula, dromedary camel production has intensified and is nowadays concentrated around cities. This may have facilitated the zoonotic ‘spillover’ infections from camels to humans, explaining the emergence of the virus in the human population in the Arabian Peninsula.

## Introduction and Method

Between 2 March 2012 and 23 July 2014, 853 people have been confirmed with Middle East respiratory syndrome coronavirus (MERS‐CoV) infections and 330 of these infections have been fatal. There is increasing microbiological evidence of dromedary camels playing a role in the zoonotic transmission of the virus, but the epidemiological evidence remains limited.

In this article, we review information about exposure of the cases and evidence of dromedary infection published before 23 July 2014, and we present possible paths of zoonotic transmission of the virus. We also explore how dromedary camel reproductive cycles and weaning could explain the seasonality of human cases, and how the shift in dromedary camel industry could explain the emergence of the virus in the Arabian Peninsula.

We performed an extensive, yet not systematic, literature review of publications since 2012 with the keywords Middle East respiratory syndrome coronavirus and MERS‐CoV. We also searched for publications and websites on camel farming, camel diseases, camel milk, camel meat and camel urine. We formed a panel of experts in public health, virology, veterinary medicine, food safety and camel production to review, validate and assess the findings. To describe the human cases, we classified cases as primary cases, cases reported as not having had documented contact with a human MERS‐CoV case, and secondary cases, household contacts of a case, cases with hospital acquired infections and healthcare workers. ECDC compiled a line listing of MERS‐CoV cases worldwide based on updates published on WHO and Ministries of Health websites around the world.

There are two species of camels: the one‐hump Arabian dromedary camel (*C. dromedarius*) that inhabits the Middle East and East Africa; and the Bactrian camel (*C. bactrianus*) with two humps that inhabits Central Asia. In this article, we focus on the Arabian dromedary camel.

## Human Cases of MERS‐CoV and Occupational Exposure

**Figure 1:**
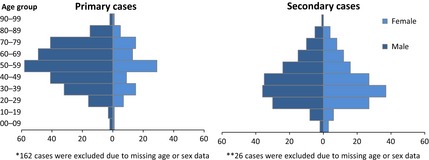
Age and sex distribution among primary and secondary cases 2 March 2012–23 July 2014 (primary cases *n* = 355*, secondary cases *n* = 310**). *162 cases were excluded due missing age or sex data. **26 cases were excluded due to missing age or sex data.ECDC line listing: data compiled from WHO and Ministries of Health websites around the world.

**Figure 2:**
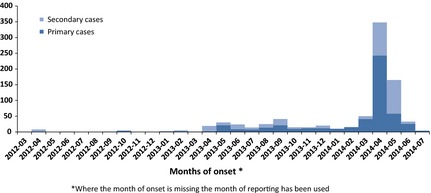
Distribution of confirmed cases of MERS‐CoV by of onset and transmission pattern, 2 March 2012–23 July 2014 (*n* = 853)*. *Where the month of onset is unknown, the month of reporting has been used.ECDC line listing: data compiled from WHO and Ministries of Health websites around the world.

Primary cases represent 61% (*n* = 517) of the reported cases. All primary cases were infected in countries of the Arabian Peninsula and more recently also in Iran; 91% (*n* = 472) of the primary cases were infected in Saudi Arabia. Men aged 40 years or above represent 58% of the primary cases (information available for 355 cases) which contrasts with the balanced age and gender distribution of the secondary cases (Fig. 1). A seasonal pattern in the temporal distribution of cases is observed; the first primary case was identified in April 2012, followed by an increase in new cases around April and May 2013 and lately a very distinct third increase in April 2014 (Fig. 2).

The cases have so far not been systematically interviewed using a standardized outbreak questionnaire, and we are concerned that the exposure to camels could be under‐reported. The lack of a standardized outbreak questionnaire also makes it difficult to estimate the true number of primary cases. It is also not unlikely that the proportion of primary cases has been over‐estimated and that more complete case information and more stringent use of case definitions would lead to substantial reclassification of primary cases to secondary cases.

Two studies measuring MERS‐CoV antibodies among camel slaughterhouse workers in Saudi Arabia and Egypt did not find evidence of previous infection in this high exposure group (Assiri et al., 2013; Aburizaiza et al., 2014) while another study in Qatar showed that people working closely with camels (e.g. farm workers, slaughterhouse workers and veterinarians) may be at higher risk of MERS‐CoV infection (World Health Organization, 2014). One study in Saudi Arabia reported that 42% of the MERS‐CoV cases had contact with camels, but the number of cases included in the study was limited (*n* = 12) (Memish et al., 2014). A review by the MERS‐CoV World Health Organization Research Group revealed that among 51 investigated primary cases, 55% (*n* = 28) reported contacts with animals but just 10% (*n* = 5) reported contacts with dromedary camels (The World Health Organization Mers‐Cov Research Group, 2013). Among 517 primary cases in the ECDC line listing, only 23 reported to have had animal contacts: 12 had direct contact with dromedary camels; 10 visited a farm (unspecified type); and 11 had unspecified contact with animals (the list is not mutually exclusive). All but one case were male with a mean age of 56 years (range 25–82). In addition, five primary cases reported drinking dromedary camel milk. None reported eating dromedary camel meat or drinking dromedary camel urine.

The age and gender distribution among primary cases is skewed towards older men, while the distribution among secondary cases is balanced. From what we have been able to establish, the gender distribution among healthcare workers in Saudi Arabia and the United Arab Emirates appears to be balanced, and the same can be assumed for household contacts of primary cases. Therefore, the imbalance among primary cases is most likely the result of differentiated exposure rather than a biological gender difference in susceptibility. It is plausible that exposure to the source of infection differs between the sexes and across age groups. Because camel rearing is an exclusively male activity that is also popular among middle‐aged and retired men, this social activity could help explain the gender difference and age‐distribution among primary cases. Both greater susceptibility to infection and higher severity among people who have underlying medical conditions (comorbidities) could help explain the higher incidence among older age groups.

A small proportion of the primary cases report having had contact with camels and camel products. The lack of systematic recording of exposure and a potential recall bias could be factors influencing these results. Animal workers, including slaughterhouse workers, do not seem to be over‐represented among MERS‐CoV cases. It is possible that animal workers develop functional immunity as a result of repetitive contacts with infected camels and therefore are at lower risk of developing severe illness. A matched case–control study looking at seroconversion rate in animal workers compared to the general population would provide valuable information.

## Evidence of Dromedary Camel Infections

MERS‐CoV infections in dromedary camels are either asymptomatic or cause mild respiratory symptoms (Hemida et al., 2014; Nowotny and Kolodziejek, 2014) suggesting that outbreaks in camel herds are likely to go undetected.

Serological studies on sheep, goats and cattle in Jordan and Saudi Arabia did not find evidence of past infection (Reusken et al., 2013a; Alagaili et al., 2014). Sera taken in 2005 in sheep and horses in the United Arab Emirates were also negative for MERS‐CoV antibodies (Alexandersen et al., 2014). The evidence incriminating bats as a source of infection is weak and unconvincing (Ithete et al., 2013; Memish et al., 2013a).

Serological studies in dromedary camels in Jordan, Oman, Qatar, Saudi Arabia and the United Arab Emirates have shown high rates of antibodies against MERS‐CoV (Reusken et al., 2013a,b; Alagaili et al., 2014; Meyer et al., 2014) indicating wide‐spread circulation of the virus in the Arabian Peninsula. Antibodies against MERS‐CoV have been detected also in dromedary camels in Egypt, Ethiopia, Kenya, Nigeria, Sudan, south Sudan, Tunisia and the Canary Islands (Perera et al., 2013; Reusken et al., 2013b; Corman et al., 2014). Retrospective analysis indicates that MERS‐CoV was circulating in dromedary camels as early as 1992 in Saudi Arabia (Alagaili et al., 2014) and 2003 in the United Arab Emirates (Meyer et al., 2014). The most recent MERS‐CoV ancestor was sampled from humans in 2011 (Rambaut, 2013).

MERS‐CoV gene fragments have been detected in nasal swabs taken from dromedary camels in Kuwait, Oman, Qatar, Saudi Arabia and Egypt (Haagmans et al., 2014; Nowotny and Kolodziejek, 2014; World Health Organisation for Animal Health, 2014), in few instances in camel faeces samples in Saudi Arabia and Qatar (Alagaili et al., 2014; Hemida et al., 2014; Reusken et al., 2014) and in camel milk in Qatar (Reusken et al., 2014). MERS‐CoV is likely to be an acute infection in camels, and there is no evidence for prolonged viral shedding beyond a few weeks. Where full genome MERS‐CoV sequences from dromedaries are available, they are highly homologous to MERS‐CoV sequences recovered from human isolates. This indicates that the same viruses can infect both camels and humans (Alagaili et al., 2014; Azhar et al., 2014; Haagmans et al., 2014; Hemida et al., 2014).

Dromedary camel calves below 1 year have lower MERS‐CoV antibody rates than older calves and adult camels (Hemida et al., 2013; Alagaili et al., 2014). Among acutely infected camels, calves below 2 years of age have been shown to have the highest viral load (Alagaili et al., 2014; Hemida et al., 2014).

Thus, there is clear evidence that dromedary camels are at least a host species for the strain of MERS‐CoV that affects humans and it is likely that primary human cases are infected through direct or indirect camel contacts. However, further epidemiological evidence and transmission studies would be useful to identify exact modes of infection to conclude that dromedary camels are the source of human infection.

## MERS‐CoV in Dromedary Camel Products and Fomites and Potential Routes of Human Infection

Camel milk consumption is marginal on a global scale, but it is increasingly popular in the Arabian Peninsula (Faye and Agricultural Research for Development, 2012). In Saudi Arabia, 78% of the camel milk production is sold as unpasteurized fresh or fermented milk to local and urban consumers (Faye et al., 2014). Cheese production from camel milk remains technically difficult, and cheese production is not a well‐developed practice (Konuspayeva et al., 2013).

MERS‐CoV was isolated in camel milk samples from mares shedding the virus (Reusken et al., 2014), although it is not clear if the virus was excreted in the milk or if the milk got contaminated during the milking process or by an infected suckling calf. When MERS‐CoV is injected into raw camel milk, the virus is stable but destroyed by heat treatment at 63°C for 30 min (van Doremalen et al., 2014). While live virus can be found in raw milk, it is still necessary to estimate virus concentrations and establish whether and for how long the virus remains infectious to humans. As a precautionary principle, consumption of raw milk and products made of raw milk should therefore be considered possible routes of infection and pasteurization of camel milk should be encouraged.

Dromedary camel meat represents 0.45% of the red meat produced worldwide (Faye, 2013; Faye et al., 2013). While there is no evidence of MERS‐CoV in camel meat, by analogy with what is known about other viruses like Rift Valley fever virus, we can assume that the fall in pH of meat with maturation could inactivate the virus (Food and Agriculture Organization) and that proper cooking would kill the virus. However, handling of raw meat and slaughtering of animals should not be excluded as a risk factor.

Washing hands, face and hair in camel urine is a traditional custom among Bedouins and camel‐herding peoples in the Arabian Peninsula and East Africa. Camel urine is also part of the traditional pharmacopoeia (Agricultural Research for Development, 2013b) and believed to have therapeutic properties for a number of ailments, including gastrointestinal conditions, and for strengthening the immune system. Fresh urine is consumed pure or mixed with camel milk, and it is also used as a component in ointments and skin creams. There are currently no published data about MERS‐CoV in the urine of infected camels, but the virus has been found in low concentration in human urine samples (Drosten et al., 2013), and therefore, consumption of camel urine may represent a risk factor for infection.

There are limited data on how long the virus survives on inanimate objects and environmental surfaces. One experiment showed that MERS‐CoV survived better than influenza A (H1N1) viruses on inanimate surfaces but less well than SARS‐CoV (van Doremalen et al., 2013). Transmission via droplets in hospital settings was demonstrated (Memish et al., 2013b) and as infected dromedary camels have very high virus concentrations in their nasal secretions, droplet spread is also a likely transmission route from camels to humans. In addition, spread via dust particles contaminated by camel urine and camel droppings cannot be excluded.

## Camel Reproduction Cycle and Weaning as Potential Factors of the Seasonality in Human Cases

Camels are seasonal breeders and calving takes mainly place between November and March in the Arabian Peninsula (Abbas et al., 2000). The first born calves are weaned in March–April which coincides with the start of the hot season. The calves are particularly susceptible to diarrhoea at this stage of their life, and mortality rates can sometimes be as high as 50% in a herd (B. Faye, unpublished data). Diarrhoea among camel calves is reported to be caused by multiple pathogens including rotavirus, coronavirus and *Salmonella* species (Al‐Ruwaili et al., 2012).

It has been observed that the epidemic peaks in human disease coincide with the start of the weaning of camel calves and the seasonal peaks in calf diarrhoea episodes. After the weaning period in the spring, the mare is milked for human consumption. If there is seasonality in the incidence among camels with a peak in the spring explained by the new birth cohort of susceptible calves, and if there is only partial immunity to MERS‐CoV among adult camels, then one would expect to see a peak in cases both among young camels and among adults. Should the virus be excreted in the milk, such a scenario could potentially lead to an increase in contamination rate of the milk during spring and consequently an increase in number of primary human cases.

Infected calves can excrete MERS‐CoV in their faeces, and the peak in diarrhoeal diseases in the spring possibly increases the exposure to infected camel faeces (Alagaili et al., 2014). Considering that milking is predominantly performed manually and potentially without proper pre‐cleaning of the teats, diarrhoeal outbreaks in calves could facilitate the introduction of virus into the milk production, either via direct contact between infected faeces and the udder or via the hands of the camel milkers. Potentially, calves can also contaminate the udder through sucking and deposit of infected saliva.

## A Shift in the Dromedary Camel Industry that May Have Facilitated the Spillover of the Virus

**Figure 3:**
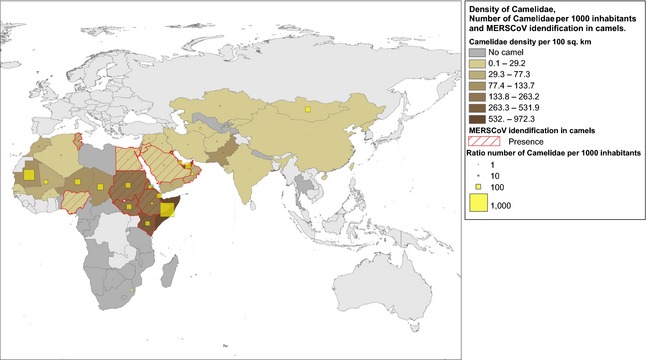
Density of Camelidae, Number of Camelidae per 1000 inhabitants and MERS‐CoV identification in camels as of 23 July 2014.The map was created using data from: World Health Organisation for Animal Health. World Animal Health Information Database (WAHID), Animal population, Camelidae, 2011–2013, Available from http://www.oie.int/wahis_2/public/wahid.php/Countryinformation/Animalpopulation. Surface area and human population: World Bank, 2009–2013. http://data.worldbank.org/. References: (Perera et al., 2013; Reusken et al., 2013a,b; Alagaili et al., 2014; Corman et al., 2014; Meyer et al., 2014).

**Figure 4:**
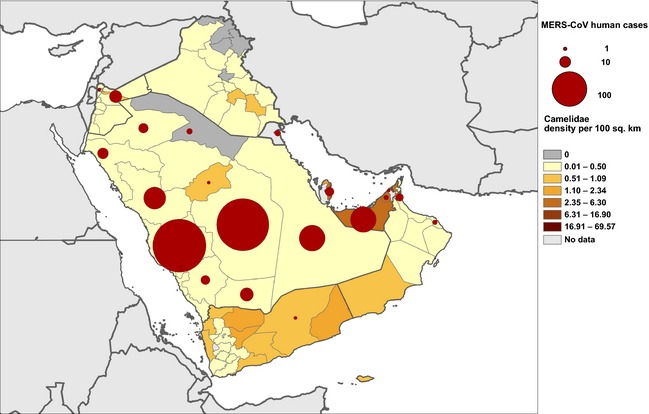
Density of Camelidae in the Arabian Peninsula and number of MERS‐CoV human cases between 2 March 2012 and 23 July 2014 (*n* = 695*). *Cases for which probable region of infection is available.The map was created using data from: World Health Organisation for Animal Health. World Animal Health Information Database (WAHID), Animal population, Camelidae, 2011–2013, Available from http://www.oie.int/wahis_2/public/wahid.php/Countryinformation/Animalpopulation. ECDC line listing: data compiled from WHO and Ministries of Health websites around the world.

The worldwide population of camels is estimated to be around 30 million, of which 95% are dromedary camels (Faye et al., 2013). Sixty percentage of the world's camels are found in the East African countries (Fig. 3) which are important camel exporters to the Arabian Peninsula and Egypt (Faye et al., 2013). There are more than 1.2 million dromedary camels in the Arabian Peninsula, and 78% of them are found in Saudi Arabia, the United Arab Emirates and Yemen (Fig. 4; Table 1) (World Health Organisation for Animal Health, 2011–2013). Qatar and the United Arab Emirates have the highest densities of camels on the Arabian Peninsula with a camel to human ratio above 0.1. On the African continent, Kenya and Somalia report the highest density of camels.

**Table 1:** Population and density per square kilometre of Camelidae in the countries of the Arabian Peninsula and African countries with over 1 million Camelidae

Country	Number of animals	Density per square 100 km	Number of camels per 1000 inhabitants
Arabian Peninsula			
Bahrain	2001	263.3	2.7
Jordan	13 501	15.1	2.1
Kuwait	35 398	18.6	12.7
Oman	131 642	42.5	37.4
Qatar	61 760	532	63.7
Saudi Arabia	213 321	9.9	7
United Arab Emirates	363 807	435.2	131.7
Yemen	370 871	70.2	15.1
African continent			
Chad	1 531 896	119.3	135.5
Ethiopia	2 245 582	203.3	29.9
Kenya	2 985 154	514.4	88.7
Mauritania	1 379 418	133.8	387.3
Niger	1 676 319	132.3	126.8
Somalia	6 200 001	972.3	624.9
Sudan	4 751 001	188.3	104.5

World Health Organisation for Animal Health. World Animal Health Information Database (WAHID), Animal population, Camelidae, 2011–2013, Available from http://www.oie.int/wahis_2/public/wahid.php/Countryinformation/Animalpopulation. Surface area and human population: World Bank, 2009–2013. http://data.worldbank.org/.

Since the 1960s, there has been a radical change in dromedary camel farming practices in Saudi Arabia. Over the past five decades, the officially reported population of dromedary camels has increased from 80 000 to more than 200 000 head and other sources estimate the true number to be as high as 800 000 (Abdallah and Faye, 2013). The dromedary camel population has been growing also in East Africa but at a slower pace.

Three farming systems can be distinguished in Saudi Arabia (Faye et al., 2014): traditional extensive farming represents about 36% of the producers and is dominated by Bedouins; semi‐intensive sedentary farming in the peri‐urban area represents 59% of the producers; and the intensive commercial farming that applies modern technologies and represents 5% of the producers.

About 20% of the dromedary camel owners are retired people and 40% are multi‐activity workers (Abdallah and Faye, 2013; Faye et al., 2014). While extensive farming is slowly declining, the other types of farming systems are growing. As a result of the rapid urbanization, Saudi camel farms have gradually concentrated around cities to respond to the increasing demand for camel milk and other camel products (Abdallah and Faye, 2013; Agricultural Research for Development, 2013a). A similar trend is observed in all the desert regions where rapid urbanization and increasing populations have increased the demand for commercial camel products (Agricultural Research for Development, 2013a). In East Africa, camel farming remains a predominantly extensive practice with the exception of Kenya and Djibouti (Faye et al., 2003).

While all primary cases so far are reported to have been infected in the Arabian Peninsula or in Iran, it is also clear that MERS‐CoV virus has been circulating for some time among camels in Africa. It is possible that the recent intensification of camel herding in the Arabian Peninsula has increased the virus' reproductive number and attack rate in camel herds while the ‘urbanization’ of camel herding increased the frequency of zoonotic ‘spillover’ infections from camels to humans.

It is reasonable to assume, although difficult to measure, that the sensitivity of public health surveillance to detect previously unknown diseases is lower in East Africa than in Saudi Arabia and that sporadic human cases may have gone undetected.

## Conclusions

There is convincing evidence that dromedary camels are host animals for the strain of MERS‐CoV that infects humans. Whether camels are indeed the reservoir for MERS‐CoV or whether they function as a vehicle for the virus from a yet unidentified animal reservoir to humans remains to be established.

Control measures implemented in the Arabian Peninsula, particularly measures to prevent healthcare associated infections, seem to be successful as the number of cases reported in June and July 2014 has drastically decreased. The critical next step is to confirm and eventually control the animal source of human infections. It is remarkable that, 2 years after the onset of this outbreak, there are still important gaps in the descriptive epidemiology of the disease. It is crucial to conduct the field epidemiology investigations that can disentangle the risk factors for infection and confirm the source of infection and the routes of transmission from the animal source to humans. A first step could be to re‐interview and reclassify all surviving case patients and the relatives of the fatal cases. This would be a time‐consuming exercise, but we believe it could generate important new hypotheses and insights that could help control this outbreak. Further microbiological experiments on milk, meat and urine should urgently be conducted to establish whether they are (effective) vehicles for virus transmission.

Public health surveillance for MERS‐CoV should be strengthened in African countries with high camel density and proofs of the circulation of the virus in camels to detect at an early stage potential human cases.

In an attempt to understand the dynamic of this outbreak, only a multisectorial approach including disciplines from the biological and social sciences will be successful. In the light of the recent increase in new cases, it is now time to manage this outbreak as the public health emergency it truly is.
